# Correction: Mukherjee et al. Insights into the Scenario of SARS-CoV-2 Infection in Male Reproductive Toxicity. *Vaccines* 2023, *11*, 510

**DOI:** 10.3390/vaccines12090996

**Published:** 2024-08-30

**Authors:** Anirban Goutam Mukherjee, Uddesh Ramesh Wanjari, Abilash Valsala Gopalakrishnan, Sandra Kannampuzha, Reshma Murali, Arunraj Namachivayam, Raja Ganesan, Kaviyarasi Renu, Abhijit Dey, Balachandar Vellingiri, D. S. Prabakaran

**Affiliations:** 1 Department of Biomedical Sciences, School of Biosciences and Technology, Vellore Institute of Technology (VIT), Vellore 632014, India; 2 Institute for Liver and Digestive Diseases, College of Medicine, Hallym University, Chuncheon 24253, Republic of Korea; 3 Centre of Molecular Medicine and Diagnostics (COMManD), Department of Biochemistry, Saveetha Institute of Medical and Technical Sciences, Saveetha Dental College & Hospitals, Saveetha University, Chennai 600077, India; 4 Department of Life Sciences, Presidency University, Kolkata 700073, India; 5 Stem Cell and Regenerative Medicine/Translational Research, Department of Zoology, School of Basic Sciences, Central University of Punjab (CUPB), Bathinda 151401, India; 6 Department of Radiation Oncology, College of Medicine, Chungbuk National University, Chungdae-ro 1 Seowon-gu, Cheongju 28644, Republic of Korea; 7 Department of Biotechnology, Ayya Nadar Janaki Ammal College (Autonomous), Srivilliputhur Main Road, Sivakasi 626124, India

The authors would like to make the following corrections to this published paper [1].

In the original publication, reference 5 was not cited along with reference 1 in the first sentence of the first paragraph in the Introduction section. The citation has now been inserted, and the sentence should read as follows after updating the order of the references:

“The coronavirus disease (COVID-19), caused by SARS-CoV-2, was first identified in Wuhan (China) in December 2019 [1,2] and has since spread fast over the world, resulting in millions of deaths and significant disruptions to healthcare systems in several countries throughout the world”. The original references 2–4 should then be re-numbered as references 3–5, respectively. 

In the sub-section “3.2. ACE2 and TMPRSS2”, the cited reference 29 should be replaced with reference 51. The sentence should read, after updating the order of the references, as:

“One study found that infertile men with substantial spermatogenesis dysfunction had lower levels of ACE2 than fertile participants [51]”.

In the sub-section “3.3. SARS-CoV-2 in Erectile Dysfunction”, the cited reference 67 should be replaced with references 2 (originally reference 5) and 66. The sentence should read, after updating the order of the references, as:

“Impairment of erectile function in COVID-19 patients might also be due to other factors, such as pulmonary fibrosis resulting in hypoxia in the penile vascular bed [2,66], a symptom of COVID-19 that might negatively affect sexual health”.

Due to the fact that the original reference 67 was removed, the following references should be re-numbered throughout the paper hereafter.

In the sub-section “3.4. SARS-CoV-2 and Oxidative Stress”, references 68 and 77 were not cited at the end of the second-to-last sentence. It should read, after updating the order of the references, as:

“SARS-CoV works via the MAPK pathway, which may trigger mitochondrial apoptotic pathways via Bax oligomerization [67,76,77]”.

In the section “6. SARS-CoV-2 and the Hormonal Changes Leading to Infertility”, the originally cited reference 69 should be replaced with references 8 and 64 in the last sentence. Therefore, the sentence should read, after updating the references:

“According to the research, ACE2-mediated pathways may be involved in how COVID-19 may affect male infertility, and infertile men may be more vulnerable to COVID-19 [8,64]”.

In the section “8. Discussions and Future Perspectives”, the cited reference 39 should be replaced with the original references 165 and 166. Therefore, the sentence should read, after updating the references and order, as:

“In a clinical study in rats, ribavirin was found to induce sperm abnormalities. This hazardous impact, however, was reversible [164,165]”. 

In Section 8, the sentence “Remdesivir is another FDA-approved drug for COVID-19 treatments that have not reported any adverse effects on the male reproductive system [168]” was incorrect and should be updated to “Comprehensive and adequately powered randomized controlled trials examining the effects of Remdesivir in hospitalized COVID-19 patients are scarce. There is insufficient evidence to support the conclusion that Remdesivir is an effective treatment for COVID-19 [167]”. The original reference 168 should be replaced with the following [2]:

Piscoya, A.; Ng-Sueng, L.F.; Parra Del Riego, A.; Cerna-Viacava, R.; Pasupuleti, V.; Roman, Y.M.; Thota, P.; White, C.M.; Hernandez, A.V. Efficacy and harms of remdesivir for the treatment of COVID-19: A systematic review and meta-analysis. *PLoS ONE* **2020**, *15*, e0243705.

In Section 8, the sentence “Various other studies focused on the role of antibiotics against COVID-19 and found that therapeutic doses of penicillin-G could lead to a spermatogenic arrest in rats after eight days of therapy [171]” was incorrect and should be updated to “A further study showed that administering therapeutic doses of penicillin G to rats for eight days led to a halt in sperm production [170,171]”. The following reference was inserted in this sentence as reference 171, and the updated references at the end of this sentence are 170 and 171 [3]:

Guo, J.; Sheng, K.; Wu, S.; Chen, H.; Xu, W. An Update on the Relationship of SARS-CoV-2 and Male Reproduction. *Front. Endocrinol*. **2021**, *12*, 788321.

The authors state that the scientific conclusions are unaffected. This correction was approved by the academic editor. The original publication has also been updated.
